# The Dietic Value of Light Wines

**Published:** 1878-10

**Authors:** 


THE DIETIC VALUE OF LIGHT WINES.—An excellent authority, Surgeon-General C. A. Gordon, of the British army, testifies strongly of the value of light wines as supplementary to insufficient food. He says, in a recent letter:—

"During the seige of Paris, not only did I, while on short food rations, experience a desire for red wine, but I felt while using it that, in a measure, it actually supplied to me the requirements of food. When the seige began and food was not very scarce, I used to take half a litre of Chablis. This I at first liked very much, but as food became scarce I found the allowance intoxicating to some degree. I then abandoned it, to drink the common *vin ordinaire*, which I enjoyed, and felt the benefit of, as I say, my allowance of it being very often, nearer a litre than half a litre. In my work on 'Army Hygiene,' I gave my views on the subject generally. What I think is very important is the absence of 'famine fever' in Paris during the seige. This was at the time, by the French medical men with whom I conversed on the subject, considered due, in a considerable degree at least, to the fact that all the population had a quarter of a litre of red wine daily." *British Medical Journal.*

